# Illinois State Veterinary Medical Association

**Published:** 1892-04

**Authors:** Matthew Wilson

**Affiliations:** Recording Secretary


*Illinois State Veterinary Medical Association.*—The semi-annual meeting of the Illinois State Veterinary Medical Association was held at Peoria, Ill , February 17th, 1892, President S. S. Baker, of Chicago, presiding. The following members responded to the roll call: Drs. Jas. Addison, Walter Allen, A. G. Alverson, S. S. Baker, G. Z. Barnes, G. L. Crocker, T. J. Gunning, Jas. McClintock, J. W. Parkinson, S. V. Ramsay, Jno. Scott, N. I. Stringer, H. Thomson, M. Wilson, N. P. Whitmore. J. A. McDonnell, M. D., of Chicago, an honorary member of the Association, was also present.

Minutes of the last meeting were read and approved. Article II, Section 3, of the by-laws, as revised at last meeting, was adopted. The following gentlemen's names were proposed for membership: Drs. Clarence Mills, (Chic. '90) Mt. Palatine; Geo. Eitewig, (Chic. '91) Canton; J. H. Malone, (Chic. '91) Henry; W. F. Robinson, (Chic. '91) Peoria; C. H. Hartman, (Am. '90) Peoria. On motion by Dr. Stringer, seconded by Dr. Scott, they were unanimously elected by acclamation.

Vice-President S. V. Ramsay taking the chair. Dr. S. S. Baker read a paper on "The Use of Lithium in Veterinary Practice." A great deal of interest was manifested in the discussion of this paper, on account of this drug being a comparatively new remedy in Veterinary practice, and by the good results following its use. The various salts of Lithium were mentioned, and the citrate chosen as being the one most applicable for veterinary use on account of its being readily dissolved. Its use was advocated in azoturia on account of its having the power of dissolving uric acid, forming a very soluble salt. It is also less irritant to the stomach than the potassium salts generally used, while its diuretic action is the same. Dr. J. A. McDonnell spoke of the good results following the use of lithium in human medicine, and advocated a more extensive use in veterinary practice. On motion the discussion was closed. The motion to adjourn until after dinner was carried.

*Afternoon session* : The President called on Dr. Whitmore, who read a paper on "Azoturia." This paper brought forth a very lengthy discussion as is usually the case when the subject is mentioned. The use of diuretics in the treatment was thoroughly discussed and favored by many, while others, from a theoretical point of view, opposed their use, on account of the already over stimulated condition of the kidneys due to the excess ofuric acid, and advocated the use of hot fermentations, the gentle stimulation of other organs, no diuretics, but soothing applications to the irritated kidneys, along with the other general treatment. A fresh kidney was brought in, and Dr. McDonnell gave a very interesting talk on the histology and physiology of this organ. The doctor was given a vote of thanks for his endeavors to benefit the Association, for the interest taken in our branch of the profession, and for his earnest work in trying to bring the two branches nearer together.

The President next called on Dr. Crocker, who gave a paper on "Nursing Sick Stock." Many points were brought out in the discussion relating to the sanitation of the surroundings of our patients, the care necessary in selecting and giving proper food, and to the giving of general directions to be carried out along with the medicinal treatment.

Dr. Alverson read a paper on "Interesting Post Mortems," and was followed by Dr. Jno. Scott with a report of two cases of "Sciatica." Two of the essayists being unable to attend, the President called for an impromptu program. Accounts were given by different members of unusual cases occurring in their practice, the results of certain forms of treatment and the outcome of rare cases. The discussions were closed on motion.

A motion by Dr. Stringer, seconded by Dr. Thomson, was made to have the chair appoint a committee, consisting of members from different parts of the State, to make out a "Fee Bill" and report at next meeting. After a good deal of discussion regarding the practicability of this plan, the motion was carried and the following committee appointed: Drs. Nattress, Scott, Cunning, Wilson, Allen, Alverson, Thomson, Whitmore and Addison. Bills to the amount of $19.25 for postage, programs, etc., were audited and ordered paid. The Treasurer's report showed a balanee of $33.32 on hand. A vote of thanks was given the proprietors of the hotel. The meeting adjourned to meet in Chicago in November, at the call of the committee.

MATTHEW WILSON, M. R. C. V. S.,
*Recording Secretary.*

